# The mental health of Vietnam theater veterans—the lasting effects of the war: 2016–2017 Vietnam Era Health Retrospective Observational Study

**DOI:** 10.1002/jts.22775

**Published:** 2022-03-15

**Authors:** Yasmin Cypel, Paula P. Schnurr, Aaron I. Schneiderman, William J. Culpepper, Fatema Z. Akhtar, Sybil W. Morley, Dennis A. Fried, Erick K. Ishii, Victoria J. Davey

**Affiliations:** ^1^ Epidemiology Program, Health Outcomes of Military Exposures (12POP5) U.S. Department of Veterans Affairs Washington D.C. USA; ^2^ National Center for PTSD U.S. Department of Veterans Affairs White River Junction Vermont USA; ^3^ Department of Psychiatry Geisel School of Medicine at Dartmouth Hanover New Hampshire USA; ^4^ VISN 2 Center of Excellence for Suicide Prevention U.S. Department of Veterans Affairs Canandaigua New York USA; ^5^ War Related Injury & Illness Study Center U.S. Department of Veterans Affairs East Orange New Jersey USA; ^6^ Population Health Services (10P4V) U.S. Department of Veterans Affairs Washington D.C. USA; ^7^ Office of Research & Development (14RD) U.S. Department of Veterans Affairs Washington D.C. USA

## Abstract

Mental health data from the 2016–2017 Vietnam Era Health Retrospective Observational Study (VE‐HEROeS) were analyzed by cohort, represented by United States Vietnam theater veterans (VTs) who served in Vietnam, Cambodia, and Laos; nontheater veterans (NTs) without theater service; and age‐ and sex‐matched nonveterans (NVs) without military service. The exposure of interest was Vietnam theater service. Surveys mailed to random samples of veterans (*n* = 42,393) and nonveterans (*n* = 6,885) resulted in response rates of 45.0% for veterans (*n* = 6,735 VTs, *M*_age_ = 70.09, *SE* = 0.04; *n* = 12,131 NTs) and 67.0% for NVs (*n* = 4,530). We examined self‐report data on four mental health outcomes: probable posttraumatic stress disorder (PTSD), depression, psychological distress, and overall mental health functioning. Weighted adjusted odds ratios (a*ORs*) between each outcome and cohort were estimated, controlling for covariates in four models: cohort plus sociodemographic variables (Model 1), Model 1 plus physical health variables (Model 2), Model 2 plus potentially traumatic events (PTEs; Model 3), and Model 3 plus other military service variables (Model 4). Mental health outcome prevalence was highest for VTs versus other cohorts, with the largest a*OR*, 2.88, for PTSD, 95% CI [2.46, 3.37], *p* < .001 (Model 4, VT:NT). Physical health and PTEs contributed most to observed effects; other service variables contributed least to a*OR*s overall. Mental health dysfunction persists among VTs years after the war's end. The present results reaffirm previous findings and highlight the need for continued mental health surveillance in VTs.

The year 1973 marked the end of American troop involvement in the Vietnam War. But for some individuals who served during this conflict, the war never really came to an end (Schlenger et al., 2015). The war lacked public support in the United States (Walker & Cavenar, 1982), and concerns associated with social support (Boscarino et al., 2018), stigmatization (Desai et al., 2016), and interpersonal and economic difficulties resonated deeply with service members transitioning back into civilian life after returning home (Boscarino et al., 2018). These adversities were compounded by war‐related traumatic experiences, which had profound effects on returning servicemembers' psychological well‐being even many years after the war (Boscarino et al., 2018).

Large‐scale epidemiological studies beginning in the 1980s have revealed the negative effects of the Vietnam War on United States veterans’ mental health. Findings from the National Vietnam Veterans Readjustment Study (NVVRS), conducted with United States veterans and civilians between 1984 and 1988 (Kulka et al., 1988a; Schlenger et al., 2015), showed that psychiatric disorders were more prevalent for Vietnam theater veterans (i.e., those with service in Vietnam, Cambodia, Laos, or the waters or airspace surrounding these countries) than for civilians, whereas high levels of exposure to war zone stress resulted in increased prevalence of disorders for Vietnam theater veterans compared with nontheater veterans or civilians (Jordan et al., 1991; Kulka et al., 1990). The Vietnam‐Era Twin (VET) Registry, developed in the 1980s (Goldberg et al., 2002), provided opportunities for further study of mental health status—male Vietnam theater veterans were shown to have poorer mental health versus their nontheater counterparts (Goldberg et al., 2016, 2014; Magruder et al., 2016).

Further examination of posttraumatic stress disorder (PTSD) was completed using NVVRS data (King et al., 1996, 1999) as well as data from the NVVRS follow‐up, the 2012–2013 National Vietnam Veterans Longitudinal Study (NVVLS; Marmar et al., 2015; Schlenger et al., 2016; Steenkamp et al., 2017). Additional work on mental health targeted female theater and nontheater veterans via the Health of Vietnam Era Veteran Women's Study (HealthViEWS; Magruder et al., 2015; Smith et al., 2020) and male, enlisted United States Army veterans of the Vietnam War via the much earlier Vietnam Experience Study (VES) (Centers for Disease Control and Prevention [CDC], 1988). The psychological health of American Legionnaires who served during the Vietnam War was also examined in the 1980s through the American Legion Vietnam Veterans Longitudinal Study, but the findings had limited generalizability because they did not constitute a representative cross‐section of Vietnam Era veterans (Stellman et al., 1988). Overall, past research may not have been representative of Vietnam theater veterans, has principally reported on PTSD, and has minimally addressed the use of nonveteran controls; use of the latter could more finely delineate the effects of service in theater on mental health. Nonveteran controls were used in the NVVRS and NVVLS research designs but not in American Legion studies, VES, HealthViEWS, or VET morbidity studies.

There has been little empirical investigation focused on statistical interactions among race/ethnicity, level of service, and mental health status in veterans of the Vietnam War; to the best of our knowledge, only one published study addressed this (Penk et al., 1989). This is an important topic because minorities’ wartime experiences, such as being marginalized or socially isolated; ethnocultural influences (e.g., perceptions of personal responsibility, coping patterns, religious beliefs; Loo, 1994; Marsella et al., 2010, 1990); and postwar socioeconomic factors may have had a more substantial impact on their mental health compared with White veterans (Dohrenwend et al., 2008; Escobar et al., 1983; Ruef et al., 2000), placing them at a potentially heightened risk of mental health disorders in later life.

Most notably, there is a diminishing opportunity to study the mental health of aging Vietnam War veterans who are in or near their 70s. Individuals in this older subset are at a period in their lives when rates of change in health status at their eighth decade of life may well differ from changes experienced in prior decades (Brayne, 2007; Gilford, 1988). Except for the NVVLS, for which slightly over 50% of all veteran respondents were at least 40 years old in 1987 at the time of the NVVRS (Schlenger et al., 2015) and were most likely approximately 65 years of age at the time of the 2012 NVVLS, there have been no other analyses of Vietnam War veterans of this kind that can offer insights into health at this later critical juncture. Questions remain regarding how theater veterans are currently functioning (Corry et al., 2016).

The present analysis used data collected by the 2016–2017 Vietnam Era Health Retrospective Observational Study (VE‐HEROeS), which was conducted to provide a broad view of the health of this older veteran population. This national, large‐scale epidemiological study was the first comprehensive health survey of United States Vietnam War veterans conducted in over 30 years. Veterans were identified using a sampling frame obtained from a well‐established database developed by the U.S. Department of Veterans Affairs (VA) and multiple other federal data sources, including the Department of Defense (DoD). VE‐HEROeS data allows researchers to compare three cohorts: (a) Vietnam theater veterans, categorized as individuals deployed to Vietnam, Cambodia, and/or Laos between February 28, 1961, and May 7, 1975, during the Vietnam War; (b) nontheater veterans, categorized as servicemembers who served elsewhere during that period; and (c) sex‐ and age‐matched United States nonveterans. Nonveteran, civilian controls were included to identify the unique effects of service in theater on health status via comparisons to theater and nontheater veterans.

The objective of the present analysis was to report on the mental health of aging Vietnam theater veterans relative to demographically comparable nontheater veterans and nonveterans. We hypothesized that psychological issues would still be prevalent nearly 50 years after the end of the war, specifically among individuals who served in the Vietnam theater, that would underscore the need for continued mental health surveillance of this population. The present analysis also included an evaluation of the relative importance of physical health, exposure to traumatic events, and other service variables on the association between cohort and mental health outcome, as well as the statistical interactions that exist among mental health outcomes, race/ethnicity, and cohort.

## METHOD

### Participants and procedure

**FIGURE 1:**
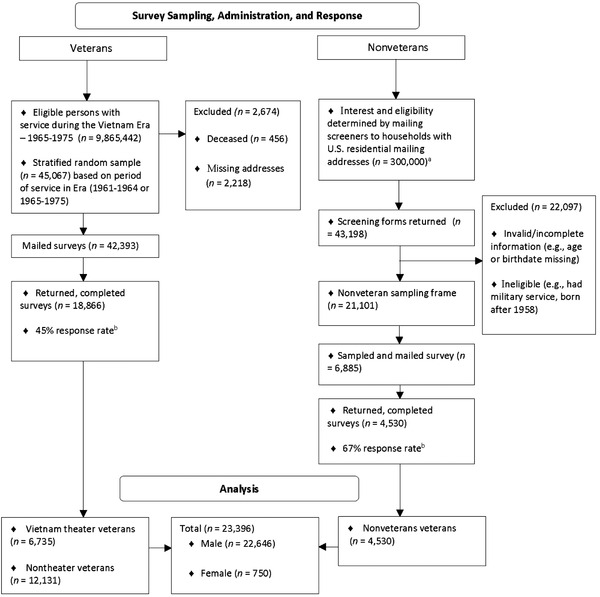
Survey flow diagram ‐ Vietnam Era Health Retrospective Observational Study *Note*. ^a^ Nonveteran sample: U.S. public general population comparison sample was an age‐ and gender‐matched sample of living nonveteran members of the U.S. public born before 1958. A two‐phase sampling approach was used based on an address‐based sampling methodology. ^b^ Based on the number of respondents, eligible nonrespondents, known ineligible individuals, individuals with unknown eligibility, and the estimated proportion of cases of unknown eligibility that were actually eligible.

The cross‐sectional 2016–2017 VE‐HEROeS was a mail survey of the health of Vietnam War theater and nontheater veterans in the United States, defined by the U.S. Congress as individuals who served in the United States military between February 28, 1961, and May 7, 1975, as well as a sex‐ and age‐matched sample of nonveterans without United States military service (Figure 1). The veteran sampling frame (*n* = 9,870,000) was derived from the VA's U.S. Veterans Eligibility Trends and Statistics (USVETS) database. Stratified random sampling was applied to the veteran sampling frame based on period of service (i.e., 1961–1964 vs. 1965–1975, periods of lowest to highest ground troop deployment) to generate the veteran sample (*n* = 45,067). Samples of 6,000 theater, nontheater, and nonveteran respondents were estimated to provide 80% power at a two‐sided significance level to detect a 20% difference in odds ratios (*ORs*) of 1.2 or greater for cohort (i.e., Vietnam theater, nontheater, nonveteran) comparisons. For the veteran sample, a 40% response rate was assumed, allowing for missing or incorrect addresses and deaths. Deceased veterans and those with missing address information were removed from the veteran sample (*n* = 2,674).

The civilian (i.e., nonveteran) sampling frame was developed in two phases that consisted of a random selection of 300,000 United States residential household addresses sourced from a commercial vendor (Phase I), after which a screener questionnaire was mailed to these households (Phase II) to identify members born before 1958 with no military service. Screener questionnaires from 43,198 households were returned, from which the nonveteran sampling frame (*n* = 21,101) was derived after removing invalid or incomplete data and ineligible individuals (e.g., had military service, born after 1958).

Main survey questionnaires were mailed to 42,393 theater and nontheater veterans and 6,885 nonveterans; all collected data were self‐reported. Questionnaires were pretested for the overall survey process and interpretability of newly developed items (Fales et al., 2019). Survey administration followed Dillman's Tailored Design Method (Dillman et al., 2014) to minimize survey error. Response rates (RRs) were 45.0% for veterans (*n* = 18,866 total, *n* = 6,735 theater, *n* = 12,131 nontheater) and 67.0% (*n* = 4,530) for nonveterans and were computed based on the number of respondents (S1), eligible nonrespondents (S2), known ineligible respondents (S3), individuals with unknown eligibility (S4), and an estimate (e) of the proportion of cases of unknown eligibility that were actually eligible, calculated as *RR* = S1/(S1+S2+e^*^S4), where e = (S1+S2)/(S1+S2+S3)] (Hintze et al., 2017). All participants received a $1 (USD) preincentive, and those who returned a completed or partially completed questionnaire received a $20 incentive. All procedures were approved by the VA‐Central Institutional Review Board, registered with ClinicalTrials.gov (#NCT02825602), and certified for confidentiality by the U.S. Department of Health and Human Service (CC‐AG‐16‐035).

### Measures

#### Mental health outcomes

##### Mental health functioning

The Mental Component Summary Score (MCS) of the Short‐Form Health Survey (SF‐8^TM^; Ware et al., 2001) was used to estimate overall mental health functioning and is one of two health‐related quality of life summary measures obtained from the SF‐8^TM^. The SF‐8^TM^ is derived from the SF‐36^®^ and is scored based on eight subscales representing physical and social health dimensions; the latter's subscales have been shown to have adequate to good internal consistency (Cronbach's αs = .78–.93; Kazis et al., 2004). In turn, the SF‐8^TM^ MCS has demonstrated high validity and test–retest reliability (i.e., *r* = .74; Ware et al., 2001), is standardized to the general population of the United States and is directly interpretable to its population mean of 50.0 (*SD* = 10). Scores of 50 or higher typically indicate average or better‐than‐average mental health–related quality of life. In this analysis, we assigned a value of 1 to indicate a below‐average score and a value of 0 to indicate an average or above‐average score. In a large subsample of VE‐HEROeS respondents (*n* = 18,056), the SF‐8^TM^ overall demonstrated excellent internal consistency, Cronbach's α = .94 (Blosnich et al., 2021).

##### 1 PTSD

The Primary Care PTSD screening measure for the fifth edition of the *Diagnostic and Statistical Manual of Mental Disorders* (*DSM*‐*5*; i.e., the PC‐PTSD‐5) was used to assess current probable PTSD. The PC‐PTSD‐5 has demonstrated high diagnostic accuracy (i.e., area under the curve [AUC] = .93, 95% CI [.90, .96]; Bovin et al., 2021) and was used dichotomously (i.e., yes/no) in the present analysis based on a recommended cutoff score of 4 (Bovin et al., 2021).

##### 2 Depression

Lifetime depression was obtained from a National Health Interview Survey (NHIS) question that was based on a provider‐identified diagnosis: “Have you ever been told by a doctor or other health professional that you had [condition]?” (CDC, 2020). The VE‐HEROeS did not collect information on current depression.

##### Psychological distress

Past‐month psychological distress, broadly defined as mental disorders such as general anxiety and mood disorders (Furukawa et al., 2003), was assessed based on the six‐item Kessler Psychological Distress Scale (K6; Kessler et al., 2003). The K6 has been shown to be a valid screening tool for serious mental illness (SMI), with good precision and discriminability (i.e., AUC = .865; sensitivity = .36, *SE* = .08; specificity = .96, *SE* = .02; Kessler et al., 2003). Scores on the six items were coded from 0 to 4 and summed, with final scores ranging from 0 to 24. K6 scores were evaluated dichotomously using a validated cutoff score of 13 or higher to indicate SMI versus 12 or lower to indicate no probable SMI (Kessler et al., 2003).

#### Other health outcomes

##### Physical health functioning

The second health‐related quality of life measure of the SF‐8^TM^ , the Physical Component Summary Score (PCS), was used to estimate physical health functioning. The PCS was assessed as a continuous variable from the same eight SF‐8^TM^ subscales underlying the MCS. The PCS has demonstrated good test–retest reliability (*r* = .73) and discriminant validity based on the SF‐36^®^ veteran version (Kazis et al., 2004; Ware et al., 2001).

##### Trauma exposure

The Brief Trauma Questionnaire (BTQ; Schnurr et al., 2002) was used to screen for 10 types of potentially traumatic events (PTEs) experienced over a lifetime, including both war‐related trauma and other trauma types. Scores were summed to obtain a count of endorsed PTE types per individual. The BTQ has been shown to have good interrater reliability (*k* = 0.89) and criterion validity (Schnurr et al., 2002).

##### Other health factors

Body mass index (BMI) was calculated as the quotient of height (kilograms) and weight (meters squared). Data regarding lifetime cigarette smoking were obtained from 2013 NHIS questions (CDC, 2020).

#### Cohort, other military service characteristics, and sociodemographic characteristics

##### 1 Cohort

The variable “cohort” comprised theater veterans, defined as individuals who served in Vietnam, Cambodia, or Laos; nontheater veterans, categorized as veterans who served during the war but not in the Vietnam theater; and nonveterans, defined as civilians in the United States who had not served in the military. Focus was placed on comparisons between theater veterans and the other two cohorts because of the recognition that Vietnam theater veterans sustained considerable health risks as part of their “Vietnam Experience” (Boyle et al., 1989). Cohort assignment came from the VE‐HEROeS survey question, “During your service, did you serve on the ground, in the air, or in the inland waters in any of the following areas between 1961 and 1975? (South Vietnam (Republic of Vietnam), Laos, or Cambodia; North Vietnam (Democratic Republic of Vietnam); Southeast Asia other than Vietnam, Laos, or Cambodia; Asia other than Vietnam or Southeast Asia; Europe; U.S.; Other).”

##### Other military characteristics

Military service branch, years of active duty service, age at entry into active duty, active duty status, Reserve status, National Guard status, the period the veteran entered active duty, and how the veteran entered service (i.e., drafted, volunteered, commissioned as an officer) were evaluated.

##### Sociodemographic characteristics

Sociodemographic variables were age at the time of survey administration, sex, educational attainment, marital status, income, and race/ethnicity. For the latter, race was categorized as White, Black, and “other race.” Non‐Hispanic White and non‐Hispanic Black were used to categorize non‐Hispanic single‐race individuals whereas non‐Hispanic “other race” was used to categorize other races of singular origin (e.g., American Indian or Native Hawaiian) and multiracial non‐Hispanic individuals. Hispanic ethnicity included participants who only reported themselves as being of Hispanic, Latino, or Spanish origin, or multiracial individuals who identified themselves as being of Hispanic, Latino, or Spanish origin.

### Data analysis

We applied SAS (SAS Enterprise Guide, Version 7.15; SAS Institute, Inc., Cary, NC, USA) procedures for complex survey designs. Statistics were weighted except for counts. Survey weights were adjusted for the complex survey design and nonresponse and poststratified using the sex and age distributions of the veteran sampling frame. To compare theater veterans to nontheater veterans and nonveterans, the nontheater and nonveteran weights were poststratified further to match the weighted age and sex distributions of theater survey respondents. Weights used in the present analysis were designed to provide prevalence estimates for Vietnam theater veterans and not for nontheater veterans or nonveterans. Jackknife repeated replication (JRR) was used to estimate standard errors based on the complex survey design. Hot deck imputation was applied to replace missing data for any one of 16 key survey questions from the total set of 82 survey items, with the estimated percentage of missing data ranging from 0.0 to 7.0%. Listwise deletion was still applied because non–key questions could include missing data. The analytical sample (*n* = 23,396, 99.7% male) consisted of 6,735 theater veterans, 12,131 nontheater veterans, 4,530 nonveterans. Women (*n* = 750) were not excluded because of the poststratification weight adjustments that were made expressly for theater veteran comparisons. Sensitivity analysis based on a domain analysis by sex revealed little or no difference in regression statistics when women were excluded (See Supplementary Table S1).

The complex design–adjusted chi‐square statistic, the second‐order Rao‐Scott chi‐square, was used to test bivariate associations between categorical variables (Heeringa et al., 2017). Tukey's test was used to control for the family‐wise error rate for multiple pair‐wise comparisons of means. Linear trend tests were conducted on mean SF‐8^TM^ scores. The prevalence of mental health outcomes was reported as a percentage with its concomitant 95% confidence interval. Crude odds ratios (*OR*s) and relative risks, the latter computed using Stata/MP (Version 15.1; See Supplementary Table S2), were computed to estimate unadjusted effect size for each of the mental health outcomes.

To evaluate adjusted effects and determine what broad categories of characteristics tended to contribute most to these effects, the association between each of the four mental health outcomes was regressed onto cohort after controlling for four categories of covariates in four models, using multiple logistic and linear regression. Model 1 included cohort plus sociodemographic characteristics (i.e., race/ethnicity, educational attainment, marital status, income), Model 2 included the covariates from Model 1 plus the physical health variables (i.e., PCS, BMI, lifetime cigarette smoking), Model 3 included the Model 2 covariates plus PTEs, and Model 4 included the Model 3 covariates plus military service characteristics (i.e., age at which the individual entered the military; military branch; active duty, Reserve, National Guard status; service length; period the veteran entered active duty; how the veteran entered service). The first‐order interaction of Cohort x Race/Ethnicity was examined. Adjusted odds ratios (a*OR*s) and unstandardized beta values with 95% confidence intervals were computed. Age and sex were not included in the models because we adjusted for them in the weights. Multicollinearity was tested, and tolerances (unweighted) above 0.40 were deemed satisfactory (Allison, 2012). We considered *p* values of less than .05 to be statistically significant.

Military service variables were added last to the models because a key goal of VE‐HEROeS was to examine the effect of cohort and other service characteristics on health. Physical health variables were added as covariates because of their associations with mental health in Vietnam‐era veterans (Beckham et al., 1998; Goldberg et al., 2014). PTEs were included to adjust for differences in lifetime traumatic event exposure. Most of the included covariates have been examined in previous mental health research among veterans of this era (Beckham et al., 1998; Boscarino et al., 2018; Goldberg et al., 2014; Kulka et al., 1990; Magruder et al., 2016; Steenkamp et al., 2017).

## RESULTS

**FIGURE 2:**
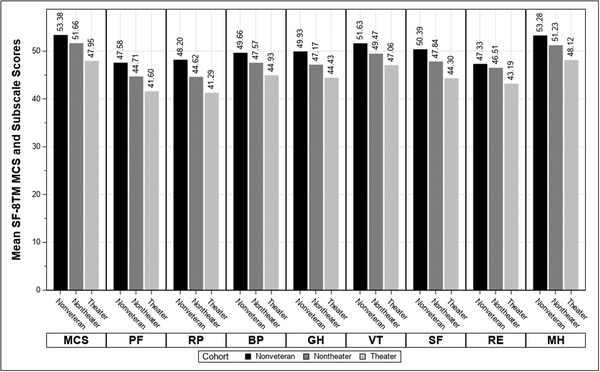
Mean SF‐8^TM^ MCS and subscale scores, by cohort *Note*. Weighted, unadjusted means are presented. MCS = SF‐8^TM^ Mental Component Summary Score; PF = physical health limits physical activities; RP = difficulty doing daily work; BP = bodily pain; GH = general health; VT = energy; SF = physical/emotional health limits social activities; RE = bothered by emotional problems; MH = emotional problems affect daily activities.

Descriptive statistics were computed for Vietnam theater veterans versus other cohorts, as shown in Table 1. The estimated mean age of Vietnam theater veterans was 70.09 years (*SE* = 0.04). SF‐8^TM^ PCS was lowest in theater veterans (*M* = 40.90, *SE* = 0.14) versus other cohorts, *p* < .001. As shown in Figure 2, a decreasing linear trend in mean MCS and all eight SF‐8^TM^ subscale scores emerged by cohort such that theater veteran scores were lowest, *p* < .001. The mean number of PTE types experienced was highest for theater veterans, *p* < .001 (see Table 1). Theater veterans were more likely to have served in the Army (54.4%), served between 1965 and 1970 (i.e., the height of the War; 73.1%), and been drafted (25.5%) compared with nontheater veterans (43.4%, 59.9%, and 19.9%, respectively).

**TABLE 1:** Characteristics of Vietnam theater veterans versus other cohorts^a^

	Vietnam theater veterans (*n* = 6,735)		Nontheater veterans (*n* = 12,131)		Nonveterans (*n* = 4,530)	
Characteristic	*n*	%	*n*	%	*n*	%
Sex						
Male	6,717	99.7	11,683	99.7	4,246	99.7
Female	18	0.3^b^	448	0.3	284	0.3
Race/ethnicity						
White non‐Hispanic	5,633	83.7	10,097	85.2	3,866	86.3
Black non‐Hispanic	487	7.1	975	7.0	244	5.9
Other non‐Hispanic	250	4.0	419	3.3	216	5.1
Hispanic	294	5.2	522	4.6	157	2.7
Educational attainment						
High school or less/GED	1,783	27.4	3,091	24.9	1,062	20.3
Some college or vocational school	2,832	43.7	5,128	41.7	1,250	24.2
Bachelor's degree or more	1,978	28.9	3,687	33.5	2,183	55.5
Marital status						
Married or with partner	5,263	79.1	9,095	78.0	3,500	79.1
Divorced, separated, or widowed	1,196	18.1	2,371	18.5	699	15.7
Never married	173	2.8	486	3.5	261	5.3
Current yearly income (USD)						
< $15,000	314	5.4	977	7.7	392	8.6
$15,000–$49,999	2,764	44.3	4,999	43.7	1,341	31.8
$50,000–$99,999	2,388	37.5	3,745	33.1	1,437	34.1
≥ $100,000	810	12.7	1,764	15.4	1,200	25.5
Lifetime cigarette smoking	4,685	71.4	8,017	66.1	2,248	53.3
Military branch						
Army	3,673	54.4	5,217	43.4	–	–
Marine Corps	827	13.0	820	5.5	–	–
Navy/Coast Guard	1,213	18.3	3,042	25.5	–	–
Air Force	998	14.3	3,003	25.6	–	–
Active duty	6,696	99.6	11,653	96.5	–	–
Reserve	1,072	16.2	2,184	21.0	–	–
National Guard	168	2.4	520	4.5	–	–
Service length (years)						
<5	4,454	69.6	8,384	74.2	–	–
5–9	591	8.9	1,272	10.0	–	–
10–19	262	3.6	412	3.3	–	–
≥20	1,362	18.0	1,518	12.6	–	–
Service period						
<1961	769	9.7	1,008	10.2	–	–
1961–1964	915	12.3	1,576	14.6	–	–
1965–1970	4,713	73.1	5,143	59.9	–	–
1971–1973	244	4.6	2,767	12.0	–	–
1974–1975	16	0.4	1,074	3.3	–	–
Entered service as						
Draftee	1,682	25.5	1,896	19.9	–	–
Volunteer	4,509	67.8	9,414	72.8	–	–
Commissioned officer	521	6.7	790	7.2	–	–

	*M*	*SE*	*M*	*SE*	*M*	*SE*
Age (years)	70.09_a_	0.04	69.86_b_	0.05	69.73_c_	0.06
BMI (kg/m^2^)	29.01_a_	0.06	28.85_a_	0.06	28.19_b_	0.10
SF‐8^TM^ PCS	40.90_a_	0.14	44.41_b_	0.11	47.88_c_	0.16
Types of PTEs experienced	2.48_a_	0.02	1.50_b_	0.01	1.13_c_	0.03
Age entered military (years)	19.93_a_	0.02	20.30_b_	0.02	–	–

*Note*: Means with different subscripts differ at the *p* < .001 level, as computed using Tukey's test. BMI = body mass index; GED = General Educational Diploma; PTEs = potentially traumatic events; SF‐8^TM^ PCS = Short‐Form Health Survey Physical Component Summary Score.

^a^ Statistics were weighted except for counts. Percentages may not add to 100% due to rounding. Associations between categorical variables were statistically significant at *p* < .001 by the design‐adjusted (second‐order) Rao‐Scott χ^2^ test. Standard errors were estimated using jackknife repeated replication.

^b^ The percentage was the same across cohorts because weights were poststratified to the sex distribution of the predominantly male, theater veteran cohort.

As shown in Table 2, the estimated prevalence of PTSD, depression, and psychological distress were higher among theater veterans than those in the remaining cohorts. Probable PTSD (18.2%, 95% CI [17.0%, 19.4%]), lifetime depression (36.4%, 95% CI [35.0%, 37.8%]), psychological distress (10.4%, 95% CI [9.5%, 11.3%]), and below‐average MCS (49.2%, 95% CI [47.8%, 50.6%]) were highest for theater veterans compared to nontheater veterans and nonveterans, *p* < .001. Estimated crude odds for PTSD among theater veterans were approximately 5–11 times the odds of PTSD among the remaining cohorts.

**TABLE 2:** Prevalence and crude odds ratios (*OR*s) for mental health outcomes^a^

	VT		NT		NV		VT: NT^b^		VT:NV^b^	
Outcome	%	95% CI	%	95% CI	%	95% CI	*OR*	95% CI	*OR*	95% CI
Probable PTSD	18.2	[17.0, 19.4]	4.5	[4.1, 4.9]	2.0	[1.4, 2.6]	4.74	[4.17, 5.38]	10.99	[7.87, 15.38]
Depression	36.4	[35.0, 37.8]	20.1	[19.5, 20.8]	15.7	[14.2, 17.1]	2.27	[2.11, 2.44]	3.07	[2.72, 3.47]
Psychological distress	10.4	[9.5, 11.3]	4.5	[4.1, 4.9]	1.8	[1.3, 2.2]	2.48	[2.18, 2.81]	6.37	[4.85, 8.40]
SF‐8^TM^ MCS^c,d,e^	49.2	[47.8, 50.6]	32.7	[31.9, 33.4]	24.4	[22.7, 26.1]	2.00	[1.87, 2.14]	2.99	[2.70, 3.33]

*Note*: VT = Vietnam theater veterans; NT = nontheater veterans; NV = nonveterans; PTSD = posttraumatic stress disorder; SF‐8^TM^ MCS = Short‐Form Health Survey Mental Component Summary Score.

^a^ Statistics were weighted, and variance estimated using jackknife repeated replication.

^b^ *ORs* were significant at *p* < .001 and unadjusted. See Supplementary Table S2 for effect sizes based on relative risk. ^c^Binary: 1 = below average score, 0 = average or above average score. Modeled below‐average score as the outcome. ^d^Continuous: *B* = −3.57, 95% CI [−3.92, −3.23], *p* < .001, for VT: NT; *B* = −4.48, 95% CI [−4.91, −4.06], *p* < .001, for VT: NV. ^e^VT: *M* = 47.95 *SE* = 0.16; NT: *M =* 51.66, *SE =* 0.08; NV: *M* = 53.38, *SE* = 0.14. Means differed at *p* < .001 (Tukey's test).

As shown in Table 3, the odds of probable PTSD, a*OR* = 2.88, 95% CI [2.46, 3.37]; depression, a*OR* = 1.57, 95% CI [1.41, 1.74]; and psychological distress, a*OR* = 1.55, 95% CI [1.32, 1.82], were significantly higher for theater veterans versus nontheater veterans after adjusting for covariates, *p* < .001 (Model 4). On average, theater veterans scored 1.23 points lower on the MCS compared with nontheater veterans after controlling for other independent variables, *B =* −1.23, 95% CI [−1.56, −0.89], *p* < .001. The effects attenuated from Model 1 to Model 4 across outcomes. The smallest decreases occurred upon the addition of other military service variables, whereas larger decreases in effects came from the addition of physical health variables and PTEs.

**TABLE 3:** Regression of mental health outcomes on cohort, controlling for various sets of covariates

	Probable PTSD		Depression		Psychological distress		SF‐8^TM^ MCS	
Model**^a^** and contrast	a*OR*	95% CI	a*OR*	95% CI	a*OR*	95% CI	*B*	95% CI
Model 1								
VT:NT	4.98	[4.35, 5.68]	2.30	[2.13, 2.49]	2.48	[2.15, 2.84]	−3.57	[−3.92, −3.23]
VT:NV	9.52	[6.85, 13.33]	2.82	[2.46, 3.23]	5.21	[3.86, 6.99]	−4.48	[−4.91, −4.06]
Model 2								
VT:NT	4.29	[3.76, 4.90]	2.01	[1.85, 2.18]	1.95	[1.71, 2.24]	−2.21	[−2.52, −1.90]
VT:NV	6.37	[4.57, 8.93]	1.99	[1.73, 2.29]	3.00	[2.19, 4.10]	−2.19	[−2.56, −1.82]
Model 3								
VT:NT	3.29	[2.83, 3.82]	1.66	[1.51, 1.82]	1.66	[1.43, 1.92]	−1.54	[−1.86, −1.23]
VT:NV	4.02	[2.87, 5.65]	1.55	[1.34, 1.79]	2.38	[1.73, 3.27]	−1.39	[−1.75, −1.02]
Model 4								
VT:NT	2.88	[2.46, 3.37]	1.57	[1.41, 1.74]	1.55	[1.32, 1.82]	−1.23	[−1.56, −0.89]
VT:NV	─	─	─	─	─	─	─	─

*Note*: All adjusted odds ratios (a*OR*s) and unstandardized regression coefficients are significant at *p* < .001. Statistics were weighted, and variance was estimated using jackknife repeated replication. PTSD = posttraumatic stress disorder; SF‐8^TM^ MCS = Short‐Form Health Survey Mental Component Summary Score; VT = Vietnam theater veterans; NT = nontheater veterans; NV = nonveterans.

^a^ Model 1: cohort and sociodemographic variables; Model 2: Model 1 plus physical health variables; Model 3: Model 2 plus potentially traumatic events; Model 4: Model 3 plus other military service variables.

As shown in Table 4, minority veterans who served in the Vietnam theater, particularly those who identified as Hispanic, generally reported higher levels of mental health dysfunction than White veterans. Among theater veterans, Hispanic participants showed significantly higher odds of reporting of PTSD, a*OR* = 3.23, 95% CI [2.33, 4.48]; depression, a*OR* = 2.08, 95% CI [1.62, 2.66]; psychological distress, a*OR* = 2.86, 95% CI [2.02, 4.03]; and lower MCS, *B* = −4.17, 95% CI [−5.11, −3.23], compared with White participants, *p* < .001. Weaker, but still significant, effects emerged for Black theater veterans versus White theater veterans for PTSD, a*OR* = 1.41, 95% CI [1.05, 1.89], *p* = .022; depression, a*OR* = 1.45, 95% CI [1.14, 1.86], *p* = .003; and MCS, *B* = −2.57, 95% CI [−3.61, −1.53], *p* < .001. Among nontheater veterans, PTSD and lower MCS were evident mostly among Black and Hispanic individuals, and PTSD was elevated among Black versus Hispanic nonveterans, a*OR* = 4.06, 95% CI [1.05, 15.65], *p* = .042.

**TABLE 4:** Associations between mental health outcomes and cohort, as a function of race/ethnicity^a^

	VT		NT		NV	
Outcome, contrast	a*OR*	95% CI	a*OR*	95% CI	a*OR*	95% CI
Probable PTSD						
Black: White	1.41*	[1.05, 1.89]	2.04***	[1.43, 2.92]	1.66	[0.50, 5.52]
Hispanic: White	3.23***	[2.33, 4.48]	1.89*	[1.09, 3.28]	0.41	[0.15, 1.15]
Other: White	1.10	[0.73, 1.65]	2.91***	[1.85, 4.57]	1.54	[0.27, 8.85]
Black: Hispanic	0.44***	[0.31, 0.62]	1.08	[0.63, 1.86]	4.06*	[1.05, 15.65]
Hispanic: Other	2.94***	[1.68, 5.15]	0.65	[0.36, 1.19]	0.26	[0.05, 1.35]
Black: Other	1.28	[0.82, 2.00]	0.70	[0.40, 1.24]	1.07	[0.12, 9.53]
Depression						
Black: White	1.45**	[1.14, 1.86]	0.95	[0.78, 1.17]	0.68	[0.40, 1.16]
Hispanic: White	2.08***	[1.62, 2.66]	0.98	[0.73, 1.32]	0.95	[0.52, 1.75]
Other: White	1.06	[0.79, 1.41]	1.27	[0.89, 1.83]	0.58	[0.25, 1.34]
Black: Hispanic	0.70*	[0.50, 0.98]	0.97	[0.67, 1.40]	0.72	[0.33, 1.55]
Hispanic: Other	1.96***	[1.32, 2.91]	0.77	[0.48, 1.23]	1.62	[0.55, 4.74]
Black: Other	1.37	[0.95, 1.98]	0.75	[0.50, 1.13]	1.16	[0.42, 3.20]
Psychological distress						
Black: White	1.19	[0.82, 1.73]	0.95	[0.65, 1.39]	1.69	[0.78, 3.66]
Hispanic: White	2.86***	[2.02, 4.03]	1.33	[0.80, 2.20]	1.19	[0.60, 2.37]
Other: White	0.73	[0.43, 1.24]	1.50	[0.82, 2.75]	2.11	[0.49, 9.09]
Black: Hispanic	0.42**	[0.25, 0.70]	0.72	[0.39, 1.31]	1.41	[0.57, 3.51]
Hispanic: Other	3.89***	[2.13, 7.14]	0.88	[0.42, 1.85]	0.56	[0.11, 2.84]
Black: Other	1.62	[0.83, 3.16]	0.64	[0.40, 1.00]	0.80	[0.14, 4.42]

	*B*	95% CI	*B*	95% CI	*B*	95% CI
SF‐8^TM^ MCS						
Black: White	−2.57***	[−3.61, −1.53]	−1.69***	[−2.44, −0.93]	−1.13	[−2.61, 0.35]
Hispanic: White	−4.17***	[−5.11, −3.23]	−1.63***	[−2.48, −0.78]	0.43	[−1.08, 1.95]
Other: White	−2.13**	[−3.42, −0.84]	−0.97	[−2.17, 0.24]	0.06	[−0.79, 0.91]
Black: Hispanic	1.60*	[0.29, 2.90]	−0.06	[−0.97, 0.86]	−1.56	[−3.71, 0.59]
Hispanic: Other	−2.04**	[−3.49, −0.58]	−0.66	[−2.05, 0.72]	0.37	[−1.25, 1.99]
Black: Other	−0.44	[−2.02, 1.14]	−0.72	[−2.21, 0.77]	−1.19	[−2.89, 0.51]

*Note*: a*OR* = adjusted odds ratio; VT = Vietnam theater veterans; NT = nontheater veterans; NV = nonveterans; SF‐8^TM^ MCS = Short‐Form Health Survey Mental Component Summary Score; PTSD = posttraumatic stress disorder.

^a^ Weighted and adjusted for sociodemographic characteristics, physical health, and potentially traumatic events. Variance estimated using jackknife repeated replication.

* *p* < .05. ^**^ *p* < .01. ^***^ *p* < .001.

## DISCUSSION

Although almost 50 years have passed since the official end of the Vietnam War in 1975, Vietnam theater veterans still report poor mental health. Adverse mental health status at this point in participants’ life trajectory was still higher for theater veterans versus nontheater veterans and nonveterans, paralleling patterns originally reported by the NVVRS in the 1980s (Kulka et al., 1990). Minority theater veterans were found to be particularly susceptible to mental health issues in older age relative to their White counterparts; this is also similar to results from the NVVRS, which was conducted when veterans from this service era were much younger (Kulka et al., 1988b, 1990). Physical health and PTE exposure were found to be important contributory factors to mental health status among theater veterans in the current analysis.

In previous studies of Vietnam‐era service members, theater veterans exposed to high amounts of war zone stress have been shown to report poorer physical health than nontheater veterans or nonveterans (Kulka et al., 1990), and individuals with PTSD have been found to report poorer physical health (Beckham et al., 1998; Goldberg et al., 2014). Associations between trauma exposure and mental health have also been found for theater veterans (Kulka et al., 1990; Maguen et al., 2009). Among VE‐HEROeS participants, an analysis of one of the BTQ items revealed that an estimated 62.9% of theater veterans experienced combat or were in a noncombat position in which they felt afraid for their lives, whereas this was reported to a much lesser extent by nontheater veterans and nonveterans (i.e., 7.1% and 1.3%, respectively), *p* < .001 (results not shown).

Combat involvement, independent of PTSD, has also been associated with poorer physical and mental health functioning in Vietnam War veterans in one study (Goldberg et al., 2014), but apart from select items from the BTQ, data on combat exposure or war zone stress were not collected in VE‐HEROeS. Moreover, although there are some related links between combat service and other military characteristics, the findings from VE‐HEROeS revealed a weak contribution of these other service characteristics to associations with these four mental health outcomes. In two studies, one of Australian male Vietnam Army veterans (O'Toole et al., 2009) and another that used data from veteran participants of the Medical Expenditure Panel Survey (O'Donnell, 2000), findings suggested that other military service variables may be less consequential to mental health than socioeconomic or physical health factors.

Data from VE‐HEROeS showed that minority veterans, particularly those with theater service, were more prone to poorer mental health status relative to nontheater veterans and nonveterans. For theater veterans, Hispanic participants were a particularly vulnerable group for whom PTSD, depression, psychological distress, and poor mental health functioning were highly evident. This is consistent with NVVRS findings showing a higher prevalence of current PTSD (27.9%) and lifetime generalized anxiety disorder (GAD) among Hispanic male theater veterans (22.4%) relative to their White counterparts (i.e., 13.7%, PTSD, 13.2%, GAD; Kulka et al., 1988b, 1990). In some cases, this occurred even after adjustment for potential predisposing variables (e.g., family background factors, health status prior to entering service) and war zone stress exposure (Kulka et al., 1990). Non‐ Hispanic Black and “other race” nontheater veterans showed evidence of mental health disorders but generally not to the same degree as Hispanic theater veterans. The higher odds of PTSD for Black nonveterans (Table 4) may reflect heightened socioeconomic disadvantage in this subgroup relative to other veterans postservice or minority nonveterans (National Center for Veterans Analysis and Statistics, 2020). Societal and cultural factors, such as racism and religious beliefs, deserve increased research attention because they could affect trauma and PTSD outcomes and should be critical components in clinical assessments and training (Marsella, 2010).

In VE‐HEROeS, the prevalence of probable current PTSD was 18.2% in theater veterans. The VE‐HEROeS estimate was higher than the PTSD Checklist for *DSM‐5* (PCL‐5) estimate (i.e., 12.2%, 95% CI [9.2%, 15.3%]) for NVVLS theater veterans (Marmar et al., 2015) as well as the 15.2% estimated for all‐male theater veterans from the NVVRS (Kulka et al., 1990). VE‐HEROeS used the five‐item PC‐PTSD‐5, whereas the NVVLS used the longer 20‐item PCL‐5, administered orally, and the NVVRS used a clinical examination composed of multiple PTSD indicators that included, for example, the Mississippi Combat‐Related PTSD Scale and Structured Clinical Interview (Schlenger et al., 1992).

In a sample of U.S. Army personnel with PTSD, a mean baseline adjusted K6 score of 10.33 was found (Olmstead et al., 2020), whereas among VE‐HEROeS participants the unadjusted weighted mean K6 scored among theater Army veterans with probable PC‐PTSD‐5 PTSD was 11.25 (results not shown). Data from the NHIS 1997–2004 revealed that the prevalence of psychological distress in adults 65 years of age and older in the United States was approximately 2%–3% (Forman‐Hoffman et al., 2014), whereas among VE‐HEROeS participants, the prevalence in similarly aged nonveterans was 1.8% and higher among nontheater and theater veterans (i.e., 4.5% and 10.4%, respectively). In the NVVRS sample, approximately 3%–11% of all theater veterans had experienced a lifetime major depressive episode, which was higher than the rates found for nontheater veterans and nonveterans (Kulka et al., 1988b); in VE‐HEROeS, lifetime depression ranged from approximately 16% for nonveterans to 36% for theater veterans.

For some theater veterans, memories of military service and later‐life events, such as the loss of family or fellow soldiers, retirement, socioeconomic concerns, weakening physical health, or cognitive decline, may have caused an emergence of late‐onset stress symptoms in older age (Davison et al., 2016; King et al., 2007). Such events may diminish resilience to renewed memories of the war, resulting in a more rapid weakening of mental and physical well‐being with increasing age (Marini et al., 2020), which is in contrast to the idea of a persistent “healthy soldier effect” (Waller & McGuire, 2011). Data from the Health and Retirement Study collected between 1992 and 2006 showed that age‐related changes in male Vietnam War veterans were not as steep as those seen in World War II or Korean War veterans (Wilmoth et al., 2010).

Several study limitations should be discussed. The results do not reflect individuals who were homeless or incarcerated and may have had a higher risk of poor mental health nor do they reflect the deceased, leaving individuals in better health to be surveyed. The VE‐HEROeS’ research design did not include oversampling minorities or women. With regard to women specifically, the findings assessed even before poststratification weight adjustments were made would not have been representative of the approximately 5,000–11,000 women veterans who served in the Vietnam theater (Earley, 1981; Thomas et al., 1991; Vietnam Veterans of America, 1985). The oversampling of women and racial/ethnic minority groups could be helpful in future studies to facilitate comparisons about these groups, especially given the changing demographic characteristics of the United States military (DoD, 2019). Prewar and postwar factors were strong predictors of PTSD symptoms (Steenkamp et al., 2017), but social support and other early‐ or later‐life factors (e.g., other military conflict involvement, health status prior to service, family environment/adverse childhood experiences; Kulka et al., 1990) were not examined. No direct measure of war zone stress or combat exposure was obtained. Retrospective and self‐reported data are prone to bias. The shorter PC‐PTSD‐5 screening instrument was administered rather than the longer PCL‐5, but the PC‐PTSD‐5 has demonstrated excellent psychometric properties (Bovin et al., 2021) and served the study's broader objectives. No measure of current depression was obtained in VE‐HEROeS; thus, we did not obtain a complete account of the current status of all four mental health outcomes. Military rank was not collected in VE‐HEROeS and would have been an important variable to examine, as findings from the NVVRS showed that across service branches, current PTSD was consistently lower among officers versus enlisted personnel (Kulka et al., 1988b). Nonveteran and nontheater veteran estimates were not representative of these respective populations because demographic characteristics were masked by weight adjustments made to make the cohorts more similar to theater veterans. Type 1 error adjustments for multiple mean comparisons were made, but there were no adjustments to alpha levels for the regression‐based comparisons. Some researchers contend, however, that these types of modifications should not be made because they may result in more data interpretation errors (Rothman, 1990).

The present study had several important strengths. Large samples of veterans and nonveteran controls provided the necessary statistical power to determine significant differences. Samples were obtained from a national frame that was generated from USVETS, an established and multisourced database of socioeconomic and benefit characteristics of all United States veterans. The veteran samples reflect a range of military service characteristics, and weights were designed to minimize bias when estimating population means and proportions for characteristics that described the theater veteran population. We examined topics with limited previous research coverage, namely the effects of military service on later‐life mental health in Vietnam theater veterans (Marini et al., 2020). Mental health patterns in later life were found to parallel findings from previous research of individuals studied at earlier points in their life course, based on findings from the VES (CDC, 1988), NVVRS (Kulka et al., 1990), VET Registry (Magruder et al., 2016), and NVVLS (Marmar et al., 2015).

In conclusion, the VE‐HEROeS adds updated information about the mental health of aging Vietnam theater veterans. The patterns of mental health disorders reported in the present analysis have persisted over decades. Overall, this study reaffirmed results from previous investigations of the mental health of Vietnam theater veterans and underscored the need for continued surveillance of the complex health effects related to involvement in the Vietnam War.

## OPEN PRACTICES STATEMENT

VA supports efforts to provide limited, restricted access to research data under written agreements consistent with commitments made to protecting subjects’ privacy and confidentiality and subject to resource availability. Contact the corresponding author for further information.

## Supporting information

Supplementary Table S1: Comparisons of Effects: Model Estimates, With/Without Women

Supplementary Table S2: Mental Health Outcomes – Relative Risks
