# Supplementary material for: The mental health of Vietnam theater veterans—the lasting effects of the war: 2016–2017 Vietnam Era Health Retrospective Observational Study
Source: J Trauma Stress. 2022 Mar 15;35(2):605–18. doi: 10.1002/jts.22775 (PMC9310606; doi:10.1002/jts.22775)
Supplement: Supplementary file 1 — Supplementary Table S1: Comparisons of Effects: Model Estimates, With/Without Women [file JTS-35-605-s002.docx]

| Supplementary Table S1 *Comparisons of Effects: Model Estimates, With/Without Women ^a^* | | | | | | |
| --- | --- | --- | --- | --- | --- | --- |
|  | With women | | | Without women | | |
| Model, comparison | *AOR*^a^ | [95% CI] | c | *AOR*^a^ | [95% CI] | c |
| Probable PTSD | | | | | | |
| Model 1 |  |  | 0.75 |  |  | 0.75 |
| VT:NT | 4.98 | [4.35, 5.68] |  | 5.00 | [4.37, 5.71] |  |
| VT:NV | 9.52 | [6.85, 13.33] |  | 9.52 | [6.85, 13.33] |  |
| Model 2 |  |  | 0.81 |  |  | 0.81 |
| VT:NT | 4.29 | [3.76, 4.90] |  | 4.31 | [3.77, 4.93] |  |
| VT:NV | 6.37 | [4.57, 8.93] |  | 6.41 | [4.57, 9.01] |  |
| Model 3 |  |  | 0.87 |  |  | 0.87 |
| VT:NT | 3.29 | [2.83, 3.82] |  | 3.30 | [2.84, 3.83] |  |
| VT:NV | 4.02 | [2.87, 5.65] |  | 4.03 | [2.87, 5.68] |  |
| Model 4 |  |  | 0.86 |  |  | 0.87 |
| VT:NT | 2.88 | [2.46, 3.37] |  | 2.89 | [2.46, 3.38] |  |
| Depression, Lifetime | | | | | | |
| Model 1 |  |  | 0.64 |  |  | 0.65 |
| VT:NT | 2.30 | [2.13, 2.49] |  | 2.31 | [2.15, 2.49] |  |
| VT:NV | 2.82 | [2.46, 3.23] |  | 2.83 | [2.48, 3.25] |  |
| Model 2 |  |  | 0.72 |  |  | 0.73 |
| VT:NT | 2.01 | [1.85, 2.18] |  | 2.02 | [1.86, 2.19] |  |
| VT:NV | 1.99 | [1.73, 2.29] |  | 2.00 | [1.74, 2.30] |  |
| Model 3 |  |  | 0.74 |  |  | 0.74 |
| VT:NT | 1.66 | [1.51, 1.82] |  | 1.66 | [1.51, 1.82] |  |
| VT:NV | 1.55 | [1.34, 1.79] |  | 1.55 | [1.34, 1.79] |  |
| Model 4 |  |  | 0.76 |  |  | 0.76 |
| VT:NT | 1.57 | [1.41, 1.74] |  | 1.57 | [1.42, 1.75] |  |

|  | With women | | | Without women | | |
| --- | --- | --- | --- | --- | --- | --- |
| Model,  comparison | *AOR*^a^ | [95% CI] | c | *AOR*^a^ | [95% CI] | c |
| Psychological distress | | | | | | |
| Model 1 |  |  | 0.72 |  |  | 0.72 |
| VT:NT | 2.48 | [2.15, 2.84] |  | 2.49 | [2.16, 2.86] |  |
| VT:NV | 5.21 | [3.86, 6.99] |  | 5.24 | [3.89, 7.04] |  |
| Model 2 |  |  | 0.81 |  |  | 0.82 |
| VT:NT | 1.95 | [1.71, 2.24] |  | 1.96 | [1.72, 2.25] |  |
| VT:NV | 3.00 | [2.19, 4.10] |  | 3.02 | [2.21, 4.13] |  |
| Model 3 |  |  | 0.83 |  |  | 0.83 |
| VT:NT | 1.66 | [1.43, 1.92] |  | 1.67 | [1.44, 1.93] |  |
| VT:NV | 2.38 | [1.73, 3.27] |  | 2.40 | [1.75, 3.29] |  |
| Model 4 |  |  | 0.83 |  |  | 0.83 |
| VT:NT | 1.55 | [1.32, 1.82] |  | 1.56 | [1.33, 1.83] |  |
| SF-8^TM^ Mental Component Summary Score | | | | | | |
|  | B^a^ | [95% CI] | R^2^ | B^a^ | [95% CI] | R^2^ |
| Model 1 |  |  | 0.13 |  |  | 0.13 |
| VT:NT | -3.57 | [-3.92, -3.23] |  | -3.58 | [-3.92, -3.23] |  |
| VT:NV | -4.48 | [-4.91, -4.06] |  | -4.49 | [-4.91, -4.07] |  |
| Model 2 |  |  | 0.30 |  |  | 0.31 |
| VT:NT | -2.21 | [-2.52, -1.90] |  | -2.22 | [-2.52, -1.91] |  |
| VT:NV | -2.19 | [-2.56, -1.82] |  | -2.20 | [-2.57, -1.83] |  |
| Model 3 |  |  | 0.32 |  |  | 0.32 |
| VT:NT | -1.54 | [-1.86, -1.23] |  | -1.55 | [-1.86, -1.24] |  |
| VT:NV | -1.39 | [-1.75, -1.02] |  | -1.39 | [-1.75, -1.03] |  |
| Model 4 |  |  | 0.33 |  |  | 0.33 |
| VT:NT | -1.23 | [-1.56, -0.89] |  | -1.23 | [-1.56, -0.90] |  |
| *Note.*  *AOR*, adjusted *OR*; c, statistic that corresponds to the area under the Receiver Operating  Characteristic curve that estimates the predictive power of a model (Allison, 2012); JRR,  Jackknife repeated replication; NT, nontheater veterans; NV, nonveterans; PTEs, potentially  traumatic events; PTSD, posttraumatic stress disorder; VT, Vietnam theater veterans. | | | | | | |
| ^a^ *AORs* and Bs are significant at the p < .001 level. Statistics were weighted. Variance  estimated using JRR. | | | | | | |
