# Supplementary material for: The mental health of Vietnam theater veterans—the lasting effects of the war: 2016–2017 Vietnam Era Health Retrospective Observational Study
Source: J Trauma Stress. 2022 Mar 15;35(2):605–18. doi: 10.1002/jts.22775 (PMC9310606; doi:10.1002/jts.22775)
Supplement: Supplementary file 2 — Supplementary Table S2: Mental Health Outcomes – Relative Risks [file JTS-35-605-s001.docx]

| Supplementary Table S2 | | | |  | | | |  | | |  | |  | |  | |  | |  | |  |
| --- | --- | --- | --- | --- | --- | --- | --- | --- | --- | --- | --- | --- | --- | --- | --- | --- | --- | --- | --- | --- | --- |
|  |  | | |  | | | |  | | |  | |  | |  | |  | |  | |  |
| *Mental Health Outcomes – Relative Risks* | | | | | | | | | | | | | | | | |  |  |  |  |  |
|  | Relative Risk^a^ | | | | | | | | | | | | | | | |  |  |  |  |  |
| Outcomes | VT:NT | | | | | | | VT:NV | | | | | | | | |  |  |  |  |  |
| PTSD, probable | 4.05 | | [3.58, 4.52] | | | | | 9.17 | | [6.18, 12.17] | | | | | | |  |  |  |  |  |
| Depression, lifetime | 1.81 | | [1.71, 1.90] | | | | | 2.32 | | [2.09, 2.55] | | | | | | |  |  |  |  |  |
| Psychological distress | 2.32 | | [2.05, 2.60] | | | | | 5.82 | | [4.26, 7.37] | | | | | | |  |  |  |  |  |
| SF-8^TM^ MCS | 1.51 | | [1.45, 1.57] | | | | | 2.01 | | [1.87, 2.16] | | | | | | |  |  |  |  |  |
| *Note:* JRR, Jackknife repeated replication; MCS, Mental Component Summary Score (1 = below average  score, 0=average/above average score); NT, nontheater veterans; NV, nonveterans; PTSD, posttraumatic  stress disorder; VT, Vietnam theater veterans  ^a^Effect size assessed via relative risks (weighted, crude) and obtained using Stata/MP 15.1 (Revision 03 Feb  2020) (StataCorp. 2017 Stata Statistical Software: Release 15. StataCorp LLC). Variance estimated using  JRR. Relative risks significant at the p < .001 level. | | | | | | | | | | | | | | | | |  |  |  |  |  |
|  |  |  | | |  |  |  | |  | | |  | |  | |  | |  | |  | |
